# Increased Flame Retardancy of Enzymatic Functionalized PET and Nylon Fabrics via DNA Immobilization

**DOI:** 10.3389/fchem.2019.00685

**Published:** 2019-10-22

**Authors:** Felice Quartinello, Klemens Kremser, Sara Vecchiato, Herta Schoen, Robert Vielnascher, Leon Ploszczanski, Alessandro Pellis, Georg M. Guebitz

**Affiliations:** ^1^Department for Agrobiotechnology (IFA-Tulln), Institute for Environmental Biotechnology, University of Natural Resources and Life Sciences, Vienna (BOKU), Vienna, Austria; ^2^Division Enzymes & Polymers, Austrian Centre of Industrial Biotechnology, Vienna, Austria; ^3^Department of Material Sciences and Process Engineering (MAP), Institute of Physics and Material Science (IPM), Vienna, Austria; ^4^Department of Chemistry, University of York, York, United Kingdom

**Keywords:** sustainable process, poly(ethylene terephthalate), nylon, enzymatic functionalization, cutinase, DNA immobilization, flame retardant

## Abstract

Poly(ethylene terephthalate) (PET) and nylon find their main applications in working clothes, domestic furniture and as indoor decoration (curtains and carpets). The increasing attention on healthy lifestyle, together with protection and safety, gained a strong interest in today's society. In this context, reducing the flammability of textiles has been tackled by designing flame retardants (FRs) able to suppress or delay the flame propagation. Commercially available FRs for textiles often consist of brominated, chlorinated and organo-phosphorus compounds, which are considered a great concern for human health and for the environment. In this study, Deoxyribose Nucleic Acid (DNA) was investigated as a green and eco-friendly alternative to halogen-containing FRs. DNA is in fact able to provide flame retardant properties due to its intrinsically intumescent building blocks (deoxyribose, phosphoric-polyphosphoric acid, and nitrogen-containing bases). In a first step, anchor groups (i.e., carboxyl groups) for subsequent coupling of DNA were introduced to PET and nylon-6 fabrics via limited surface hydrolysis with *Humicola insolens* cutinase (HiC). Released monomer/oligomers were measured via HPLC (1 mM of BHET for PET and 0.07 mM of caprolactam from nylon after 72 h). In a next step, DNA immobilization on the activated polymers was studied by using three different coupling systems, namely: EDC/NHS, dopamine, and tyrosine. DNA coupling was confirmed via FT-IR that showed typical bands at 1,220, 970, and 840 cm^−1^. The tyrosine/DNA coupling on nylon fabrics resulted to be the most effective as certified by the lowest burning rate and total burning time (35 s, 150 mm, and 4.3 mm^*^s^−1^ for the blank and 3.5 s, 17.5 mm, and 5 mm^*^ s^−1^ for nylon/tyrosine/DNA) which was also confirmed by FT-IR and ESEM/EDS measurements. Thermogravimetric analysis (TGA) further confirmed that tyrosine/DNA coated nylon showed a lower thermal degradation between 450 and 625°C when compared to the untreated samples.

**Graphical Abstract:**
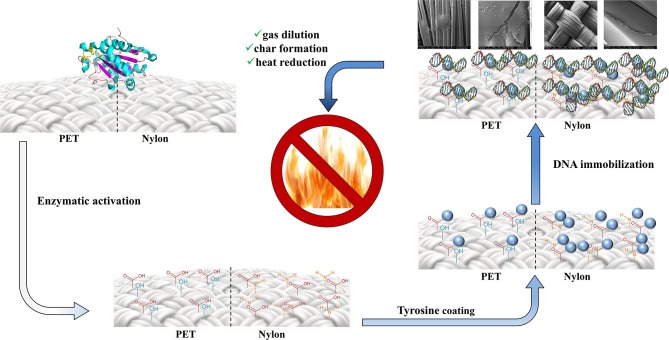
Two-step procedure for the production of environmentally-friendly DNA-based materials with flame retardant properties.

## Introduction

Synthetic polymers, like poly(ethylene terephthalate) (PET) and nylon-6, are omnipresent in an average household (furniture, wall insulation, curtains, flooring, etc.) as well as in clothing. Within European fire brigade statistics, specially referring to these kinds of fibers and plastics, different studies confirmed that they greatly contribute to a quick flames spreading in case of fire, leading to major damages to humans and also high financial losses (World Fire Statistics, 2006). For this reason, flame retardant science gained a crucial role in textile and plastic manufacture in the last decades. Briefly, the application of Flame Retardant Compounds (FRC) prevent the ignition or lead to the self-extinguishing of the flame. FRC are added to the polymer surface as result of pretreatment (Vecchiato et al., 2017b) or as post treatment (Chen et al., 2015). The most widely used FRC are represented by inorganic or organic compounds, containing metal ions or halogenated atoms. Among the inorganic FRC, aluminum trihydrate (AlH_6_O_3_) and magnesium hydroxide (Mg(OH)_2_) are the most prominently used in ceramic surface treatment. During the fire propagation, these compounds have the property to release water during their endothermic degradation (Wang et al., 2017). Within the class of halogenated flame retardants, chlorine and bromine-based compounds are the most widespread used because they are able to prevent the further heat propagation and thermal combustion (Horrocks and Price, 2001). The last class of flame retardants comprises phosphorus-based flame retardants, often combined with nitrogen compounds. During the fire, these compounds release phosphoric acid (which leads to char formation) and ammonia (which causes a dilution in the gas phase) (Wang et al., 2018). However, in the last years, several hazard impact studies of such FRC were reported, and together with the strict directives from EU community led to the ban of several halogenated FRC, since they show high toxicity for both human and animals (Salmeia et al., 2016). As demonstrated by Watanabe (Watanabe and Sakai, 2003), the major emission of brominated and chlorinated compounds occurs directly during production. Moreover, the amount of landfilled and incinerated textiles per years also constitutes a problem for the environment (Quartinello et al., 2018). Most of the FRC are absorbed on surface material, therefore their leaching and consequently accumulation, cause major health risks also for marine environment and in the end also for human health due to their dietary.

The development of new and less toxic alternative FRC is definitively required. It has been demonstrated that natural compounds, like phytic acid and cyclo-dextrine (Feng et al., 2011; Cheng et al., 2016) may act as promising eco-friendly FRC for poly(lactic acid). Casein and hydrophobins have also shown FR potential, due to their phosphoserine and cysteine content in their structures (Alongi et al., 2014), releasing phosphoric acid, ammonia and sulfuric acid, which are able to reduce the fire spreading.

The DNA's double helix consists of two single strands of nucleotides. Each nucleotide contains one of the four nitrogen-containing bases adenine, guanine, cytosine, or thymine. Together with a deoxyribose unit and a phosphate group they built up the sugar phosphate backbone of the DNA. The two antiparallel single strands are connected via hydrogen bonds to form the well-known α-helical structure with minor and major groove in between. Several studies have already proven the potential of DNA as flame retardant for example on cotton and wool fabrics using a layer by layer absorption (Alongi et al., 2013). By comparing DNA with the commercially available flame retardants, DNA provides all desired requirements to be used as a flame-retardant compound (Bosco et al., 2015). The nitrogen present in adenine, guanine, cytosine, and thymine as well as deoxyribose units acting as carbon source, have the potential to release ammonia and carbon dioxide upon heating, which are non-combustible gases and can lead to self-extinguishing by displacing the oxygen. Furthermore, the sugar-phosphate backbone can release phosphoric acid during combustion, which leads to degradation and char formation and in further consequence to the barrier of the combustible material (Alongi et al., 2013). The availability of DNA has already become competitive to those of other chemicals as described by Grote et al., which developed a large-scale method for the extraction of DNA out of salmon milt and roe sacks in waste products of Japanese fishing industry (Grote et al., 2005). Furthermore, alternative sources of waste like spend brewer's yeast (Ferreira et al., 2010) and vegetable waste (Yang et al., 2007) have been proven to be potential sources for DNA and can present a cheap and sustainable alternative to chemical flame retardants. Another advantage of using DNA is that a high purity is not necessary because also impurities like proteins can have positive effects due to their nitrogen content.

In this work, we investigated a covalent immobilization of DNA onto enzymatically activated PET and nylon-6. Therefore, after enzymatic, mild surface activation of both polymers, DNA was coupled using three different “natural-inspired” approaches, in order also to reduce the amount of chemical and biohazards compounds for human health and for the environment.

## Materials and Methods

### Chemicals, Substrates, and Enzymes

Poly(ethylene terephthalate) (PET) and polyamide 6 (nylon 6) fabrics, at thickness of 0.41 ± 0.015 mm and weight of 240 ± 12 g(m^2^)^−1^ were purchased from Goodfellow (Huntingdon, UK). All the other chemicals and solvents were purchased from Sigma-Aldrich (Vienna, Austria) at reagent grade and used without any further purification if not specified. The *Humicola insolens* cutinase (HiC) (CAS 9000-62-1) was purchased by Novozymes (Copenhagen, Denmark), and used without purification steps.

### Biochemical Characterization of HiC

Protein concentration was determined with the BIO-RAD Protein Assay Protocol (Bio-Rad Laboratories GmbH, Cat. No: 500-0006) using bovine serum albumin (BSA) as protein standard. Specifically, 10 μL of protein standard solution were added into the well of a 96 well plate (Greiner 96 well plate bottom transparent polystyrene). Afterwards, 200 μL of 1:5 diluted Bio-Rad reagent were added. The plate was incubated for 5 min at 21°C and 300 rpm. The buffer (100 mM potassium phosphate pH 7) was used as blank. Relative protein absorption was measured at λ = 595 nm, and the concentration was evaluated from the average of triplicates samples and blanks. Esterase activity assay was performed using *p*-nitrophenyl butyrate (*p*-NPB) as model substrate, as previously reported by Biundo et al. (2016). The increase of absorbance at 405 nm due to the releasing of *para*-nitrophenol was measured for 5 min, every 18 s with a plate reader (TECAN INFINITE M200). A blank was considered using 20 μL of buffer instead of enzyme solution. The activity was defined in unit (U), where 1 unit is considered as the amount of enzyme necessary to hydrolyze 1 μmol of substrate per minute under the given assay conditions.

### Enzymatic Functionalization of PET and Nylon 6

#### Enzymatic Functionalization

Prior to the enzymatic treatment, PET and nylon fabrics were cut into 1 cm^2^ pieces with a hot wire and washed according to a commonly used washing protocol. Briefly, samples were washed with a Triton-X 100 solution (5 g/L w/v) followed by a washing step with Na_2_CO_3_ (100 mM) and mQ-H_2_O as the last step. The complete washing procedure was performed at 60°C, 30 min, and 150 rpm on a thermo-shaker. Finally, samples were dried for 24 h at 21°C (Vecchiato et al., 2017a).

Fabric samples were incubated in triplicates with three different concentrations of HiC (0.1, 0.25, and 0.5 mg/mL). The reaction was performed in 5 mL Eppendorf Tubes in a total volume of 4 mL potassium phosphate buffer (100 mM, pH 7). Enzymatic hydrolysis was performed for 24, 48, and 72 h at 150 rpm and 60°C. After the incubation, samples were washed according to the washing protocol and dried for 24 h at 21°C. For the upscale set up, samples with a size of 8 × 20 cm were incubated with an enzyme concentration of 0.25 mg/mL in a 2 L Erlenmeyer shaking flask for 72 h at 150 rpm at the same temperature.

To detect carboxyl (-COOH) and amino (-NH_2_) groups on the surface of PET and nylon-6 fabrics after the enzymatic hydrolysis, a colorimetric method, using two different color reagents, was tested. For the determination of -COOH groups, the basic dye Methylene Blue (MB) (Figure S1A) was used. Samples with the size of 1 × 1 cm were incubated with 1.5 mL MB (0.5% in mQ-H_2_O) at 80°C for 60 min. After incubation, samples were washed according to the washing protocol and dried for 24 h at 21°C (Song and Kim, 2017). To detect -NH_2_ groups on the surface of nylon, samples were treated with the acid dye Coomassie brilliant blue (CBB) (Figure S1B). The samples were incubated in 1.5 mL of CBB (0.5 mg/mL) in acid solution (mQ-H_2_O/methanol/acetic acid 85:10:5 v/v, pH 2.2) for 5 min at 21°C. To wash off the unbound dyes, samples were intensively rinsed with acid solution (Noel et al., 2011).

In order to determine the difference in color intensity of the PET and nylon samples after the treatment with acid/basic dye, a ColorLite sph850 Spectrophotometer—Color Measuring Instrument (Innovac, Germany) was used. This allows determination of the color difference between the blank (no enzymatic treatment) and the treated samples using the L*, a*, and b* coordinates. The interpretation of the measurement is based on the theory defined by the Commission Internationale de I‘Eclairage (CIE), that two colors cannot be red and green or yellow and blue on the same time. The coordinates L*, a*, and b* indicate the lightness, red/green, and yellow/blue difference. Enzymatic treated PET and nylon samples were measured in triplicates (5 measurements per sample) against the non-treated blanks. To determine the color difference, the delta (Δ) L*, a*, and b* should be calculated.

$$\begin{array}{l} \;\Delta {\text{L}}^{\wedge }{}^{*}=(\text{L}\_{\text{sample}}^{\wedge }{}^{*}-\text{L}\_{\text{blank}}^{\wedge }{}^{*})\\ {\left(\Delta \text{a}\right)}^{\wedge }{}^{*}=(\text{a}\_{\text{sample}}^{\wedge }{}^{*}-\text{a}\_{\text{blank}}^{\wedge }{}^{*})\\ {\left(\Delta \text{b}\right)}^{\wedge }{}^{*}=(\text{b}\_{\text{sample}}^{\wedge }{}^{*}-\text{b}\_{\text{blank}}^{\wedge }{}^{*}) \end{array}$$

The values for ΔL*, a*, and b* can be positive or negative. ΔL* indicates the difference in lightness (+ = lighter, – = darker), Δa* the difference in red and green (+ = redder, – = greener) and Δb* the difference in yellow and blue (+ = yellower, – = bluer).

#### High Performance Liquid Chromatography (HPLC)

The release products after enzymatic hydrolysis of PET and nylon were measured via HPLC. To remove protein impurities from the sample, a methanol precipitation step was performed with ice cold methanol (1:1 sample/methanol) (Quartinello et al., 2017). Afterwards, samples were centrifuged at 12700 rpm for 15 min. at 4°C (5920 R Centrifuge from Eppendorf) followed by an acidification step with 6 N HCl (6 μL per sample). Before pipetting the samples into the HPLC-vials, a filtration step with a 0.45 μM PA filter was performed. For the measurements of the PET released product (Ta), an Agilent LC-MS system was used with a Poroshell 120 column (InfinityLab Poroshell 120 EC-C18, 4.6 × 50 mm, 4 μM, Agilent), a flow of 0.35 mL/min and a non-linear gradient (Table S1). The released products were detected at 241 nm via UV-Vis spectroscopy. Caprolactam released from nylon-6 was measured using an Agilent LC-MS system with a phenomex® column (Aqua® 5 μm C18, 125 Å, LC Column, 250 x 4.6 mm) with an isocratic gradient (H_2_O/MeOH, 60/40 [% v/v]) and flow of 0.5 mL/min for 50 min. Hydrolysates were measured at 210 nm via UV-Vis spectroscopy.

### DNA Immobilization on Surface Activated Polymers

#### DNA Characterization

To determine the length of the DNA molecules, an agarose gel electrophoresis was performed. A 3% agarose gel was produced by dissolving 1.2 g of agarose in 40 mL of 1x TAE-Buffer (50x Tris/Acetic Acid/EDTA (TAE), diluted with distilled deionized water) by heating in a microwave oven. For staining of the DNA, 4 μL nucleic acid gel stain were added to the gel. After casting the gel, it was allowed to solidify for 30 min and was put into a Mini-Sub cell GT cell from BIO RAD. For the preparation of the DNA samples, 5 μL of a DNA loading dye were added to 25 μL of sample. Twelve microliters of the samples and 5 μL of Orange Ruler 10 bp DNA ladder (Thermo Fischer, USA) were pipetted on the gel and allowed to run for about 30 min at 120 V. Gels were afterwards imaged using a ChemiDocTM MP imaging system from BIO RAD. For the measurement of the DNA content, an Implen NanoPhotometer® was used. Therefore, the photometer was blanked, using only MES-buffer and afterwards the samples were measured in triplicates at 260 nm.

#### DNA Cross-Linking Methods

To find the right concentration of DNA, DNA was dissolved in MES-Buffer (2-(N-morpholino)ethanesulfonic acid, 100 mM, pH 4.6) at 150 rpm and room temperature for 3 h, with 7% DNA solutions. The linkage between the salmon DNA and the pre-treated PET or nylon 6 samples was performed in three different ways using 1-Ethyl-3-(3-dimethylaminopropyl)carbodiimide (EDC)/N-Hydroxysuccinimide (NHS), dopamine or tyrosine as crosslinking agents (Figure S2).

##### EDC/NHS cross-linking

For the crosslinking of DNA using the EDC/NHS system, an EDC stock solution with 200 mg/mL and a NHS stock solution with 400 mg/mL were produced in MES-Buffer (100 mM, pH 4.6) (Pieper et al., 2000). Crosslinking reactions were performed in 12-well plates in a volume of 3 mL at 150 rpm and 21°C. MES-Buffer, EDC, NHS, and DNA stock solutions were added in suitable amounts to reach final concentrations in the reaction mixture of 50 mg/mL EDC, 100 mg/mL NHS and 7% DNA. At first the right amount of MES buffer was added to the fabric, followed by 750 μL EDC stock solution. After 10 min, 750 μL NHS stock were added and after another 10 min incubation time, 1,260 μL (7%) of DNA stock solution was pipetted to the reaction mixture. The DNA was allowed to bind for 24 h at 250 rpm and 21°C. After incubation, samples were dried for 24 h at 21°C.

##### Dopamine/tyrosine coupling

To use dopamine or tyrosine as crosslinking agents, a 2 mg/mL dopamine hydrochloride solution and a 2 mg/mL tyrosine solution were prepared in Tris/HCl buffer (100 mM, pH 8.5). For the coating with either dopamine or tyrosine, 3 mL of the solutions were pipetted to enzymatically treated PET and nylon-6 samples and incubated for 24 h at 250 rpm and 21°C. After incubation, samples were washed by dipping them 3 times in mQ-H_2_O. DNA immobilization reaction was performed with a 7% DNA solution (in MES-buffer) which was directly pipetted to the dopamine or tyrosine samples. The incubation with DNA was performed for 24 h at 250 rpm and 21°C. After the incubation, the samples were dried for 24 h at room temperature (Lee et al., 2009).

### Fourier-Transformed Infrared Spectroscopy (FT-IR)

To measure changes on the surface of PET and nylon after enzymatic treatment and the different coating steps, a PerkinElmer Spectrum 100 FT-IR Spectrometer was used. Spectra were recorded from 4,000 to 650 cm^−1^. A total of 25 scans for each sample were recorded with a resolution of 2 cm^−1^. Normalization of the recorded spectra was done at the band occurring in the 1,410 cm^−1^ region (CH in plane bending and CC stretching) for PET and in the 1,460 cm^−1^ (-CH_2_ bending vibration) for Nylon, which has been already proven to be a suitable reference band (Donelli et al., 2009). The bands were assigned as follows: for PET, 1,471 cm^−1^ (-CH_2_ bending), 1,410 cm^−1^ (-CH in plane bending and CC stretching), 1,340 cm^−1^ (-CH_2_ wagging), 1,120 cm^−1^(-O-CH_2_ and ring CC stretching), 970 cm^−1^ (-O-CH_2_ and C=O stretching), 847 cm^−1^(bending of benzene rings. For nylon: 3,300 cm^−1^ (-NH stretching and bending vibration of secondary amine), 1,640 cm^−1^ (-NH bending of Amide I), 1,540 cm^−1^ (-C=O stretching of Amide II), 1,460 cm^−1^ (-CH_2_ bending vibration), 1,250 cm^−1^ (-C-N stretching vibration), 960 and 930 cm^−1^ (-CH out of plane bending). For DNA: 1,680 cm^−1^ (P=O), 1,220 and 1,060 cm^−1^ (symmetric and asymmetric PO^2−^ vibration), 967 cm^−1^ (phosphodiester bond), 832 and 782 cm^−1^ (sugar-phosphate bond) (Socrates, 2004).

### Environmental Scanning Electron Microscopy (ESEM)

For the SEM investigations a FEI Quanta 250 FEG (Thermo Fisher Scientific, Hillsboro, OR) was used under high vacuum condition and a variable high tension from 5 to 10 kV. The micrographs were recorded with the Everhart-Thornley-Detector in secondary electron (SE) mode. The fracture surface was sputtercoated with a 10 nm thin layer of gold in order to provide sufficient electrical conductivity. The EDS measurements were performed for 60 s, with 20 kV high tension and a Spotsize of 4,5 with a 10 mm^2^ Apollo X Silicon Drift Detector by EDAX Ametek, NJ. USA, and Genesis Software V 6.53 from 21 September, 2018.

### Durability of Washing

In order to confirm the covalent binding of DNA on fabrics a washing stability test was performed. Briefly DNA coated samples (8*20 cm) were washed in a solution containing 4 g/L of commercial detergent and the liquor ratio was 50:1. Each step was conducted at 40°C for 5 min. After each wash, the fabric was removed, squeezed and rinsed with mQ-H_2_O. The washing tests were repeated till 100 min were reached. After each washing step the DNA concentration present in solution was measured via Implen NanoPhotometer® as described above. The percentage of DNA was calculated from the initial (or previous) DNA content, and compared with absorbed DNA on untreated and enzymatic treated fabrics.

### Flame Retardant Assessment

#### Flammability Test

Flammability tests were done according the ISO 6940 for the determination of ease of ignition of vertically oriented specimens. Samples were cut in the size of 8 × 20 cm and fixed in the apparatus (Figure S3) (Kozlowski et al., 1989). For the ignition of the sample, the flame of the burner was set to a vertical height of 4 cm. Tests were performed in bottom edge ignition, in which the flame was applied to the sample to the shortest time to cause ignition. The whole experiment was recorded on video and the time was measured from the time of ignition until self-extinguishing was reached or the sample completely burned. This test gives information about the burning time, burning length and the resulting burning rate. Furthermore, one can gain information about self-extinguishing, char-formation and the ease of ignition. An increase in flame-retardancy would result in longer burning time, a decrease of the burning length and a lower burning rate. Samples were weighted before and after the flammability test, in order to quantify the char formation.

#### Thermogravimetric Analysis (TGA)

TGA was performed on a PL Thermal Sciences STA 625 thermal analyzer, using 10 mg of sample in an aluminum pan. The gas flow (N_2_ or O_2_) was set to 100 mL^*^min^−1^ and samples were heated from 30 to 625°C at a heating rate of 10°C^*^min^−1^. The temperatures at 5 and 50% mass loss (TD_5_ and TD_50_) were subsequently determined.

## Results

### Enzymatic Functionalization

Polymer-degrading enzymes represent useful tools for specific but mild introduction of reactive chemical groups on polymer surfaces, retaining their bulk properties (Pellis et al., 2016). Different lipases and cutinases have demonstrated to be able to functionalize polyesters in a first instance (Gamerith et al., 2017; Ortner et al., 2017). On the other hand, *Fusarium solani* cutinase was also applied for hydrolysis of polyamides (Silva et al., 2007) leading that cutinases are able also to partly modify nylon surfaces.

*Humicola insolens* cutinase (HiC) had a protein concentration of 10 mg/mL and specific activity of 80 U/mg against *p*-PNB. To determine the optimal concentration of the enzyme for functionalization, different concentrations (0.1, 0.25, and 0.5 mg/mL) were incubated with 1 cm^2^ fabric pieces (PET and nylon) at 60° C. For PET functionalization, the total amount of terephthalic acid released showed the highest concentration (0.05 mM) with 0.5 mg/mL of HiC after 72 h. On the other hand, the higher concentration of caprolactame (2.2 μM released from nylon) was obtained with 0.25 mg/mL of enzyme after 72 h.

**Figure 1:**
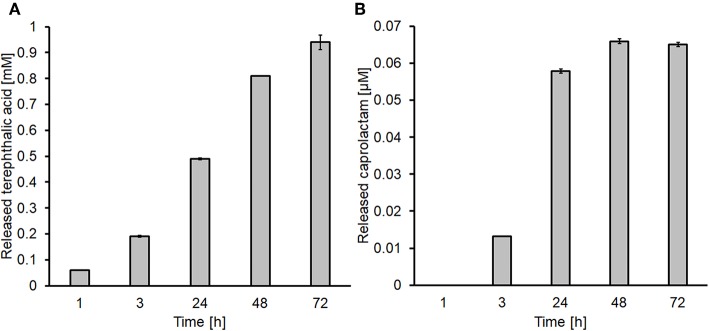
Determination of the released products after enzymatic hydrolysis via HPLC. **(A)** Terephthalic acid released from PET and **(B)** caprolactam released from nylon-6.

These optimal conditions were further used for the functionalization of 8^*^20 cm stripes of both fabrics. The amounts of released products were 1.0 and 0.07 mM for terephthalic acid and caprolactam, respectively (Figure 1).

The application of acid and basic dyes, confirmed the presence of newly formed functional groups on polymer surface, based on changes of L^*^, a^*^, b^*^, and E^*^ compared to the blanks (Figure S4). The delta (Δ) values of PET indicated that samples became darker, greener and bluer (Table 1). The same trend was found for polyamide, but with lower extent. This confirmed the HPLC results, with the lower amount of caprolactame released, but nevertheless more reactive groups were generated via enzymatic treatment.

**Table 1:** Color changes of PET and nylon surface before and after enzymatic treatment.

	L*	a*	b*	ΔE*
**METHYLENE BLUE (ACID DYE)**				
Untreated PET	134.15	8.75	31.70	0.06
PET_HiC	101.38	7	28.27	4.8
Delta (Δ)	−2.77	−1.75	−3.43	4.74
**COMASSIE BRILLIANT BLUE (BASIC DYE)**				
Untreated nylon	73.04	−1.31	−8.44	0.02
nylon_HiC	66.36	−1.42	−9.55	−8.59
Delta (Δ)	−6.68	−0.11	−1.11	8.57

**Figure 2:**
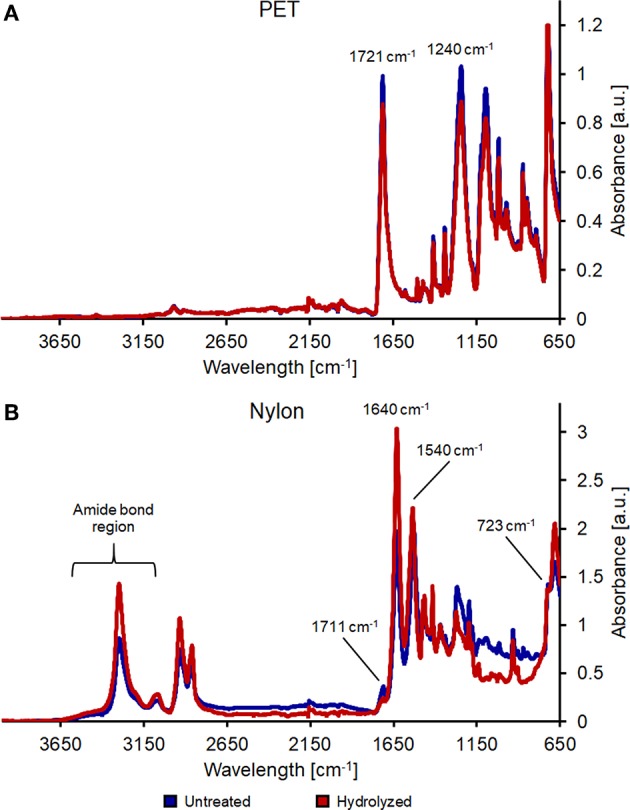
FT-IR analysis of untreated (blue line) and enzymatically hydrolyzed (red line) PET **(A)** and nylon-6 **(B)**.

Further proofs of the presence of more functional moieties were obtained via FT-IR measurements. The enzymatic hydrolysis of PET showed a reduction and a slight shift of the band at 1,721 cm^−1^ (indicative of the carbonyl stretching group). Moreover, the band at 1,240 cm^−1^ (C=O-O stretching and CC stretching) was lower, confirming that the enzyme cleaved some esters bonds within the PET fibers (Figure 2A).

Nylon spectra show instead differences in the 3,300–2,800 cm^−1^ region, indicating partial breakage of amide bonds. Additionally, bands at 1,640 and 1,540 cm^−1^ decreased, while bands at 1,711 cm^−1^ (C=O vibration) and 723 cm^−1^ (-NH_2_ vibration) clearly indicated the formation of new carboxylic and amine groups (Figure 2B).

### DNA Immobilization

Low molecular weight Deoxyribose nucleic acid (DNA) from salmon sperm was used. Agarose gel defined that the length of DNA molecules were in the 20–30 bp size range (Figure S5). This size is suitable for immobilization applications since longer DNA fragments could hamper the reaction progression due to steric hindrance. As preliminary test, FT-IR of salmon DNA was recorded to discriminate DNA bands after the immobilization on PET and nylon-6. DNA characteristics bands resulted to be at 1,680 cm^−1^ (P=O) with the presence of a small shoulder (C=O vibration of guanine, cytosine, and thymine), 1,220 and 1,060 cm^−1^ (symmetric and asymmetric PO^2−^ vibration, and as well band at 967 cm^−1^ (phosphodiester) and 832, 782 cm^−1^ for sugar-phosphate bond (Figure S6).

#### EDC/NHS Coupling System

The EDC/NHS coupling system is a multistep reaction able to link the carboxylic groups with primary amines. In a first step, EDC reacts with the obtained carboxylic groups on PET and nylon surface, leading the formation of acylisourea intermediate, which in a second step reacts with NHS to form an amino reactive sulfo-NHS ester. This last compound is then replaced by DNA molecule due to a stable amine bond with carboxylic acid group. Functionalized PET spectra after DNA immobilization using EDC/NHS as coupling system showed only minor changes: in particular, it was possible to observe an increase of the peak at 1,710, 1,230, and 1,090 cm^−1^ characteristic from the DNA molecule (Figure S7). In enzymatically activated nylon-6, the band at 1,630 cm^−1^ was intensified and shifted into the direction of P=O band of DNA. Moreover, changes in intensity of the peak in the area of 1,060–960 cm^−1^ are typical from phosphate or phosphate ester moieties, indicating that the nucleic acid was immobilized in small extend (Figure S8).

#### Dopamine and Tyrosine as Cross-Linkers

Dopamine is a small catecholamine, precursor of norepinephrine, and epinephrine (brain neurotrasmettitors). In nature these compounds are used by mussels for the attachment to rock surfaces, mainly due to the ability to form thin polydopamine layers on surfaces (Rollett et al., 2013). FT-IR spectra of PET after dopamine coating were recorded, showing broad and intensified bands between 3,600 and 2,900 cm^−1^ due to hydrogen bonds which are formed between hydroxyl groups and amino groups from dopamine. Intensified bands in the 1,650–1,450 cm^−1^ area due to N-H bending vibrations and aromatic C-C stretching vibrations clearly confirm the presence of dopamine after coating (Luo et al., 2014). Further characteristic peaks of dopamine are 1,265 cm^−1^ (aromatic amine C-N stretching vibrations) and 1,050 cm^−1^ (C-N bending vibrations between the benzene ring and the hydroxyl groups) that in the spectra are overlapping with bands characteristic for PET. In PET coated with dopamine/DNA, a new band at around 1,680 cm^−1^ (P=O) appeared, as well an intensified and shifted bands at 1,220 cm^−1^ and 1,060 cm^−1^ (asymmetric and symmetric PO^2−^ vibrations), which clearly indicate the presence of immobilized DNA. This is also confirmed by intensified and shifted bands at 835 and 780 cm^−1^ which contribute to C=C and C=N stretching modes of pyrimidines and purines (Figure S9) (Alongi et al., 2013).

Dopamine coating of nylon samples showed changes in the FT-IR spectra, which correspond to the ones recorded for PET/Dopamine. Bands with a broader band within the range of 2,800–3,600 cm^−1^ indicate the formation of hydrogen bonds between the hydroxyl groups of dopamine. The band at 1,493 cm^−1^, characteristic for aromatic C-C stretching vibrations of dopamine, is overlaid by the amide II band of nylon but there is a shift into the direction of dopamine. A brighter and intensified peak at ~1,265 cm^−1^ as well as a clearly visible peak at around 1,050 cm^−1^ further confirms the presence of dopamine on the surface of nylon fabrics. Spectra of nylon/Dopamine/DNA samples showed an intensified, shifted peak which also forms a small shoulder at ~1,540 cm^−1^. Furthermore, a sharper and more intensified peak was recorded between 1,470 and 1,420 cm^−1^. A shift of the band at ~1,006 cm^−1^ and a broader bandwidth of the peak at around 835 cm^−1^ which are characteristic for C=C and C-N stretching modes, again confirm that also the DNA immobilization on dopamine coated nylon fabric was performed (Figure S10).

On the surface of nylon fabric, dopamine forms scurf-like particles which are attached to the fibers (Figure S11). There is no homogenous coating of DNA visible which is also confirmed by the FT-IR spectra, showing only low bands in the characteristic region of DNA. Imaging of PET after Dopamine/DNA coating showed again the scurf formation of dopamine on the fiber. In both fabrics, DNA coating was a non-homogenous layer (Figure S11).

**Figure 3:**
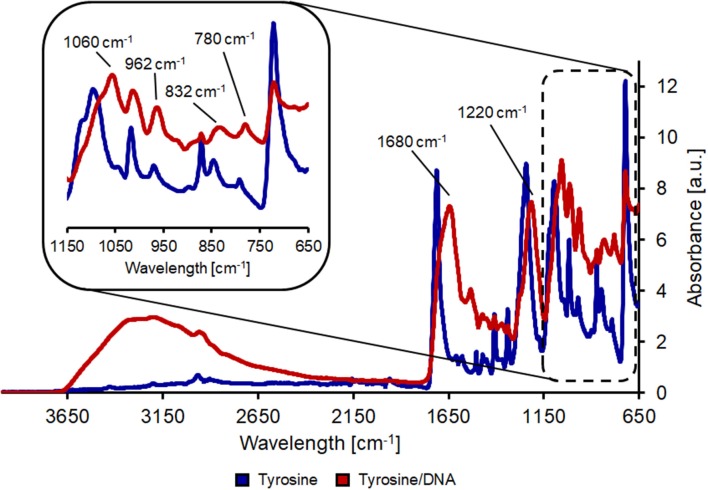
FT-IR analysis of hydrolyzed PET after coating with tyrosine (blue line) and after tyrosine/DNA coupling (red line).

L-tyrosine is a proteinogenic amino acid, also precursor of L-Dopa. Its chemical structure provides two reactive group (an amino and a carboxylic groups), which are suitable for cross-linking binding of macromolecules, such as DNA. In the spectrum from PET/tyrosine, bands at 3,210 and 3,040 cm^−1^ (aromatic CH stretching), 1,590 cm^−1^ (asymmetric stretching of ${\text{NH}}_{3}^{+}$) and 1,450 cm^−1^ (scissoring vibration of tyrosine) are visible after the treatment (Wang et al., 2016). By comparing the spectra of PET/tyrosine/DNA with the spectra of salmon DNA all peaks characteristic for DNA (1680, 1220, 1060, 967, 832, and 780 cm^−1^) are clearly visible, which confirms the presence of DNA on the PET fabric. Due to the intense and clearness of the bands it was proven that DNA was immobilized on the polymer's surface (Figure 3).

**Figure 4:**
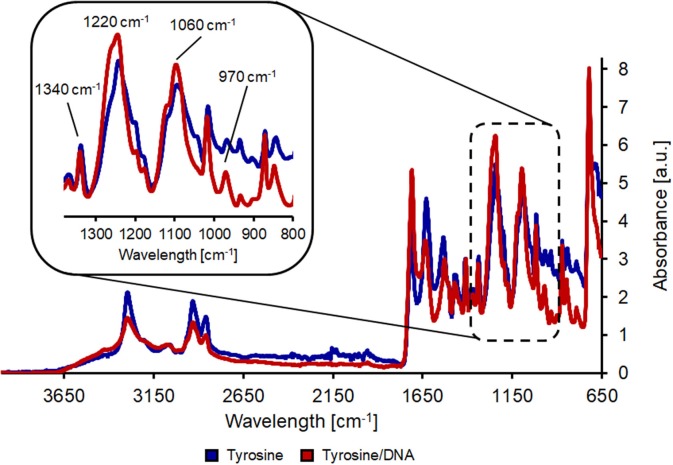
FT-IR analysis of hydrolyzed nylon-6 after coating with tyrosine (blue line) and after tyrosine/DNA coupling (red line).

The nylon/tyrosine reaction also showed distinct changes in the FT-IR spectra when compared to the blank. As described earlier, the reduction of the amide I and II bond (1,657 and 1,533 cm^−1^) and the increase in the band at 1,711 cm^−1^ is due to the hydrolysis of the polymer bonds which leads to an increase in carboxylic acid groups. This is also the reason for the intense band at ~1,100 cm^−1^ which results from newly formed carboxyl groups. Nevertheless, tyrosine characteristic peaks were also visible in the case of nylon/tyrosine. These peaks are again located at 3,040, 1,590 (overlaid by the amide II band), and 1,330 cm^−1^. Furthermore, there is a very strong and wide peak at 1,250 cm^−1^ (OH in plane deformation coupled to C-O stretching) and together with the peak at 840 cm^−1^ the spectra clearly confirm that _L_-tyrosine is coupled to nylon fabric. The band at 1,710 cm^−1^ was intensified and shifted toward the direction of the P=O bonding after the immobilization of DNA. Furthermore, bands at 1,220 and 1,060 cm^−1^ were further intensified and show a slight shift. At ~970 cm^−1^ the intensity of the band decreases which is due to ribose phosphate skeletal motions (Alongi et al., 2013). The shift of the bands at 1,410 and 1,340 cm^−1^ can be explained by the presence of purine and pyrimidine molecules (1,470–1,320 cm^−1^) (Figure 4).

**Figure 5:**
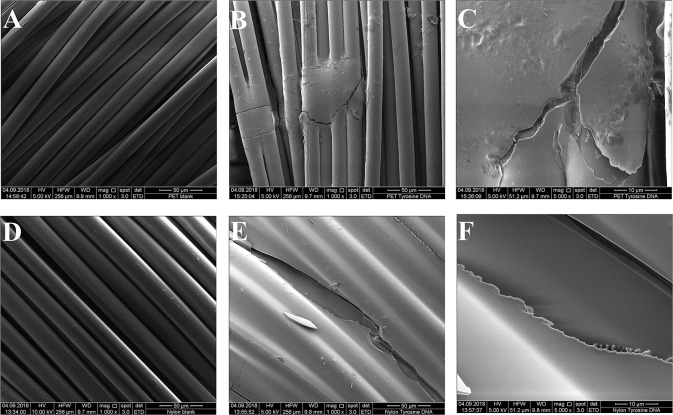
Scanning electron microscopy images of the samples. **(A)** Untreated PET 1000x magnification **(B)** PET_tyrosine_DNA 1000x magnification **(C)** PET_tyrosine_DNA 5000x magnification **(D)** untreated nylon 1000x magnification **(E)** nylon_tyrosine_DNA 1000x magnification **(F)** nylon_tyrosine_DNA 5000x magnification.

**Figure 6:**
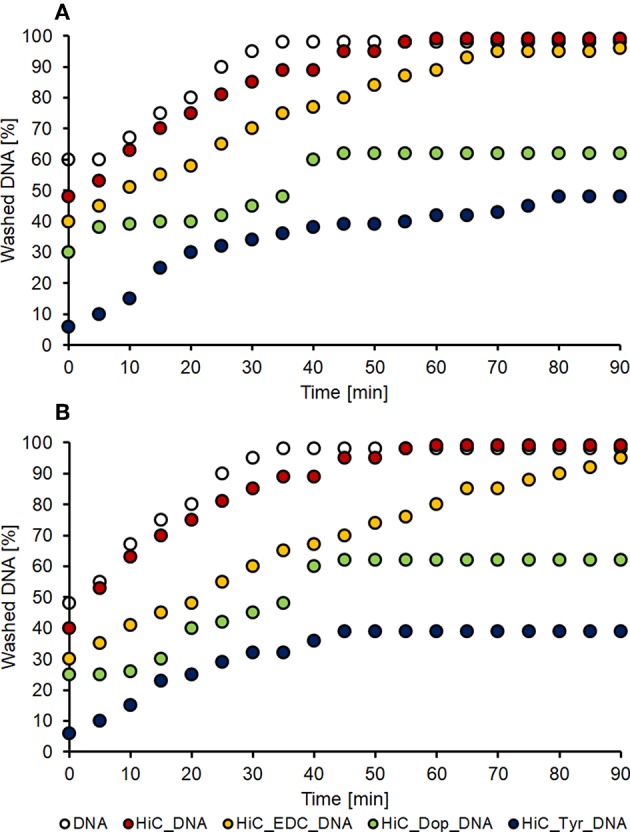
DNA washing stability. Washing of PET **(A)** and nylon **(B)** samples treated with DNA absorbed on untreated polymer (white dots), DNA absorbed on enzymatic treated polymer (red dots), and the three coupling conditions: EDC coupling (orange dots), dopamine coupling (green dots) and tyrosine coupling (blue dots).

**Figure 6** shows the clear surface structure differences of tyrosine and DNA coated samples. For tyrosine coating, crystals were formed on the surface, while DNA covered the fibers homogeneously. This was confirmed by the EDS spectra, showing a newly formed and intense peak of phosphorus (Figure S12). Compared to Nylon, the DNA-immobilization on PET was not homogenous, showing spots of DNA distributed on the surface of the sample. Furthermore, the EDS spectra showed a smaller peak for phosphorus compared to nylon/tyrosine/DNA (Figure 5).

The washing stability tests confirmed the previous results: the samples treated with EDC/NHS as cross-linking method showed the same DNA leaching trend as well the control samples, indicating that DNA was mostly absorbed. On the other hand, the dopamine and tyrosine coating systems demonstrated great covalent immobilization stability; around 50% and 40% of DNA, respectively, were leached out after 45 min washing and stable (Figure 6).

Summarizing, FT-IR, SEM and washing step results, EDC/NHS coupling lead to a low for DNA immobilization of PET and nylon fabrics. Using Dopamine/DNA was not homogenously attached onto two polymer and moreover dopamine coating gave a dark brownish color to both textiles, which can prohibit a potential textile application. Finally, tyrosine coating exhibited the optimal condition for DNA immobilization, stability and color, and was therefore applied for further flame retardant tests.

### Flame Retardant Tests

For polyester, the three treatments showed a decrease in burning rate compared to the untreated (blank) and enzymatic treated (HiC) samples. The total burning time (TBT) increased as well as the length of the burned sample, showing that the immobilization of the DNA favors the flame-retardancy of PET fabrics (Table 2). Tyrosine/DNA treated PET samples resulted in a decreased burning rate and length of the burned specimen.

**Table 2:** Flammability of PET and nylon fabrics after enzymatic surface activation and coupling of DNA.

**Sample**	**Total burning time [s]**	**Burning length [mm]**	**Burning rate [mm/s^**−1**^]**
PET Blank	6.75	57.5	8.5
PET_HiC	5	42.5	8.5
PET_HiC_tyrosine_DNA	11	68	6.2
nylon Blank	35	150	4.3
nylon_HiC	10	60	6.0
nylon_HiC_tyrosine_DNA	3.5	17.5	5.0

**Figure 7:**
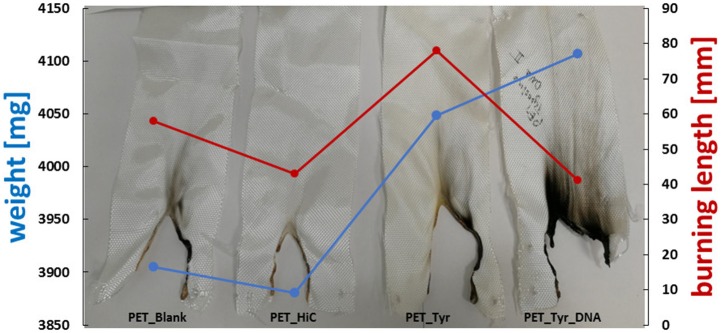
Results of flammability tests of enzymatically activated and tyrosine and tyrosine/DNA treated PET.

An increase in the char formation during burning of the tyrosine/DNA treated sample also indicates the presence of DNA (Figure 7). Compared to the untreated and enzymatically treated sample, it was possible to decrease the burning rate from 8.5 to 6.2 mm^*^s^−1^, which further confirms that DNA provides flame retardant properties. Moreover, PET/blank showed a melting behavior of the fibers during combustion. In contrast, the tyrosine/DNA coated samples showed more intact fibers as well as char formation due to still attached DNA which serves as protection layer during thermal decomposition of the polymer.

**Figure 8:**
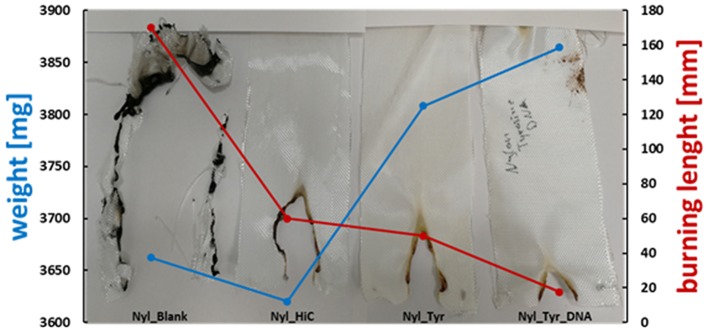
Results of flammability tests of enzymatically activated and Tyrosine and Tyrosine/DNA treated nylon.

Nylon fabric showed different behavior compared to PET. Starting with the untreated nylon fabric which completely burned and did not manage to self-extinguish the flame (longest TBT), there is a clear trend in decreasing length of the burned sample visible throughout the different coating steps. In contrast, the enzymatic treated (nylon_HiC) fabric showed an about 1/3 reduced TBT and burning length. This may be explained by the newly formed amine groups after enzymatic hydrolysis, which can release ammonia during combustion, leading to a dilution in the gas phase and an extinguishing of the flame. Tyrosine/DNA seems to be the best treatment also for nylon fabric, due to the shortest TBT (few seconds) and burning length (~18 mm), furthermore, the flame was self-extinguished nearly immediately after the flame was applied to the fabric (Figure 8). After coating with tyrosine and immobilization of the DNA, the flame was immediately self-extinguished after ~18 mm and a few seconds. The brownish color of theburned parts, which results from sugars within the sugar-phosphate backbone of DNA, confirms the presence of DNA on the nylon fabric.

**Figure 9:**
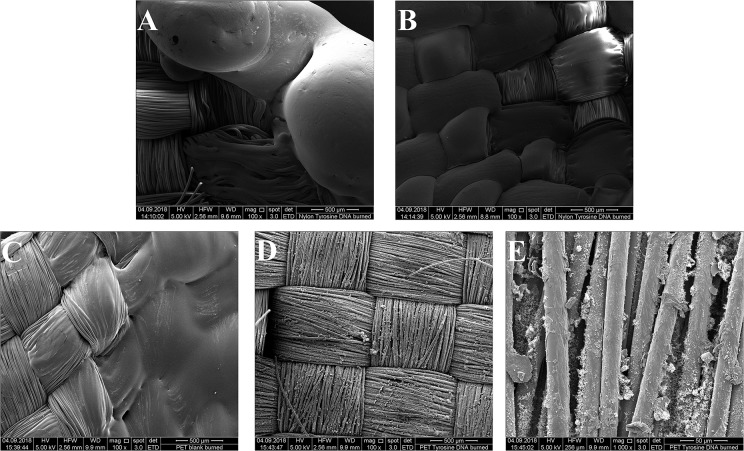
ESEM image of Nylon/Blank burned (100x) **(A)** and Nylon/tyrosine/DNA burned (100x) **(B)** in the upper part and PET/Blank burned (100x) **(C)**, PET/tyrosine/DNA burned (100x), **(D)** and PET/tyrosine/DNA burned (1000x) **(E)**.

In case of Tyrosine/DNA coated samples, the swelling behavior of DNA during combustion indicates the release of non-combustible gases, leading to the very quick self-extinguishing of the flame. Beneath the DNA coating, nylon fibers are still intact and have not been attacked by the heat. PET/Blank showed a melting behavior of the fibers during combustion. In contrast, the tyrosine/DNA coated samples showed more intact fibers as well as char formation due to still attached DNA which serves as protection layer during thermal decomposition of the polymer (Figure 9).

**Figure 10:**
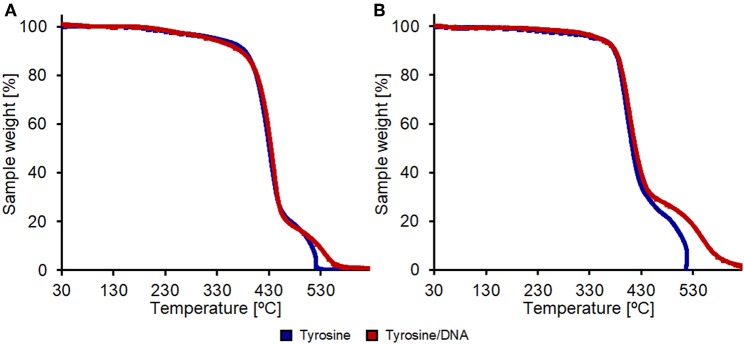
Thermal degradation analysis in air of PET **(A)** and nylon **(B)** samples. All samples were enzymatically hydrolyzed and coupled with only tyrosine (blue lines) or with tyrosine and DNA (red lines).

Thermogravimetric analysis performed under an air flow shows changes in thermo-oxidative behavior and thermal degradation while the same analysis performed under a N_2_ flow didn't lead to any significative changes between the various samples (Figures S13, S14). Tyrosine/DNA-coated PET showed a slower thermal degradation in the 500–600°C region, indicating that DNA favors the flame-resistance (Figure 10A).

In the case of nylon, the coating with Tyrosine and DNA showed even higher effects in terms of thermal resistance. The weight-loss due to thermal degradation between 450 and 625°C was significantly lower compared to the nylon sample treated with only tyrosine that shows a complete weight loss at 516°C (Figure 10B). This further confirms that the DNA-immobilization on nylon was more successful if compared with the one performed on PET fabrics.

## Conclusions

DNA provides all properties of a flame-retardant compound (temperature reduction by the release of water, release of inert gases and formation of a solid layer), has been investigated to be an eco-friendly alternative to commercial FR in terms of availability and the rather mild operation conditions. Enzymatic surface activation of PET and nylon prior to DNA coupling was successful via *Humicola insolens* cutinase, as confirmed by HPLC, dyes application and FT-IR. To immobilize DNA on the surface of PET and nylon, three different crosslinking agents have been applied (EDC/NHS, Dopamine and tyrosine), in which tyrosine has shown to be the most sufficient. This was also confirmed by the FT-IR spectra which present the most intense bands of DNA after the coating with L-tyrosine as well as the ESEM images showing homogeneous coating. These results were successfully proved with the flame retardant test, where this coating coated fabrics shown the lowest TBT and burning length.

## Data Availability Statement

The datasets generated for this study are available on request to the corresponding author.

## Author Contributions

FQ, KK, HS, and RV performed the experiments. FQ, AP, SV, and GG planned the experiments. LP performed SEM measurements. FQ and AP wrote the manuscript. AP and GG supervised the work. All authors discussed the collected data and corrected the manuscript before submission.

### Conflict of Interest

The authors declare that the research was conducted in the absence of any commercial or financial relationships that could be construed as a potential conflict of interest.

## References

[B1] Alongi J. Carletto R. A. Bosco F. Carosio F. Blasio A. D. Cuttic F. (2014). Caseins and hydrophobins as novel green fl ame retardants for cotton fabrics. Polym. Degrad. Stab. 99, 111–117. 10.1016/j.polymdegradstab.2013.11.016

[B2] Alongi J. Carlettob R. A. Blasioa A. D. Carosioa F. Boscob F. Malucellit G. (2013). DNA: a novel, green, natural flame retardant and suppressant for cotton. J. Mater. Chem. A 1, 4779–4785. 10.1039/c3ta00107e

[B3] Biundo A. Hromic A. Pavkov-Keller T. Gruber K. Quartinello F. Haernvall K.. (2016). Characterization of a poly(butylene adipate-co-terephthalate)-hydrolyzing lipase from Pelosinus fermentans. Appl. Microbiol. Biotechnol. 100, 1753–1764. 10.1007/s00253-015-7031-1 26490551

[B4] Bosco F. Casaleab A. Molleaa C. Terlizzic M. E. Gribaudoc G. Alongi J. (2015). Surface & coatings technology DNA coatings on cotton fabrics : effect of molecular size and pH on fl ame retardancy. Surf. Coatings Technol. 272, 86–95. 10.1016/j.surfcoat.2015.04.019

[B5] Chen S. Li X. Li Y. Sun J. (2015). Intumescent flame-retardant and coatings on cotton fabric. Surf. Coatings Technol. 262, 4070–4076. 10.1021/acsnano.5b00121 25777158

[B6] Cheng X.W. Guan J. P. Tang R. C. Liu K. Q. (2016). Phytic acid as a bio-based phosphorus fl ame retardant for poly (lactic acid) nonwoven fabric. J. Cleaner Prod. 124, 114–119. 10.1016/j.jclepro.2016.02.113

[B7] Donelli I. Taddei P. Smet P. F. Poelman D. Nierstrasz V. A. Freddi G. (2009). Enzymatic surface modification and functionalization of PET: a water contact angle, FTIR, and fluorescence spectroscopy study. Biotechnol. Bioeng. 103, 845–856. 10.1002/bit.22316 19365872

[B8] Feng J. Su S. Zhu J. (2011). An intumescent flame retardant system using b -cyclodextrin as a carbon source in polylactic acid (PLA). Polym. Adv. Technol. 22, 1115–1122. 10.1002/pat.1954

[B9] Ferreira I. M. P. L. V. O. Pinhoab O. Vieiraa E. Tavarela J.G. (2010). Brewer's *Saccharomyces* yeast biomass: characteristics and potential applications. Trends Food Sci. Technol. 21, 77–84. 10.1016/j.tifs.2009.10.008

[B10] Gamerith C. Gajda M. Ortner A. Acero E. H. Guebitz G. M. Ulbricht M. (2017). Enzymatic hydrolysis of poly(ethyleneterephthalate) used for and analysed by pore modification of track-etched membranes. New Biotechnol. 39, 42–50. 10.1016/j.nbt.2017.06.007 28698130

[B11] Grote J. G. Heckman E. M. Diggs D. E. Hagen J. A. Yaney P. P. Steckl A. J. (2005). DNA-based materials for electro-optic applications: current status. SPIE 5934:593406 10.1117/12.615206

[B12] Horrocks A. R. Price D. (2001). Fire Retardant Materials. Elsevier.

[B13] Kozlowski R. Mieleniak B. Muzyczek M. Fiedorow R. (1989). Flammability and flame retardancy of leather. 1989, 1–9.

[B14] Lee H. Rho J. Messersmith P. B. (2009). Facile conjugation of biomolecules onto surfaces via mussel adhesive protein inspired coatings. Adv. Mater. 21, 431–434. 10.1002/adma.200801222 19802352 PMC2755254

[B15] Luo B. Wang X. Wang Y. Li L. (2014). Fabrication, characterization, properties and theoretical analysis of ceramic/PVDF composite flexible films with high dielectric constant and low dielectric loss. J. Mater. Chem. A 2, 510–519. 10.1039/C3TA14107A

[B16] Noel S. Liberelle B. Robitaille L. De Crescenzo G. (2011). Quantification of primary amine groups available for subsequent biofunctionalization of polymer surfaces. Bioconjug Chem. 22, 1690–1699. 10.1021/bc200259c 21736371

[B17] Ortner A. Pellisb A. Gamerith C. Yebra A. C. Scaini D. Kaluzna I. (2017). Activated poly (lactic acid) surfaces. Green Chem. 19, 816–822. 10.1039/C6GC03150A

[B18] Pellis A. Herrero Acero E. Ferrario V. Ribitsch D. Guebitz G. M. Gardossi L. (2016). The closure of the cycle: enzymatic synthesis and functionalization of bio-based polyesters. Trends Biotechnol. 34, 316–328. 10.1016/j.tibtech.2015.12.009 26806112

[B19] Pieper J. S. Hafmans T. Veerkamp J. H. van Kuppevelt T. H. (2000). Development of tailor-made collagen-glycosaminoglycan matrices: EDC/NHS crosslinking, and ultrastructural aspects. Biomaterials 21, 581–593. 10.1016/S0142-9612(99)00222-7 10701459

[B20] Quartinello F. Vajnhandl S. Volmajer Valh J. Farmer T. J. Vončina B. Lobnik A.. (2017). Synergistic chemo-enzymatic hydrolysis of poly(ethylene terephthalate) from textile waste. Microbial Biotechnol. 10, 1376–1383. 10.1111/1751-7915.12734 28574165 PMC5658601

[B21] Quartinello F. Vecchiato S. Weinberger S. Kremenser K. Skopek L. Pellis A.. (2018). Highly selective enzymatic recovery of building blocks from wool-cotton-polyester textile waste blends. Polymers 10:1107. 10.3390/polym10101107 30961032 PMC6403871

[B22] Rollett A. Thallinger B. Ohradanova-Repic A. Machacek C. Walenta E. Cavaco-Paulo A. (2013). Enzymatic synthesis of antibody-human serum albumin conjugate for targeted drug delivery using tyrosinase from Agaricus bisporus. RSC Adv. 3, 1460–1467. 10.1039/C2RA22560C

[B23] Salmeia K. A. Gaan S. Malucelli G. (2016). Recent advances for flame retardancy of textiles based on phosphorus chemistry. Polymers 8:319. 10.3390/polym8090319 30974592 PMC6432008

[B24] Silva C Araújo A. Casal M. Gübitzc G. M. Cavaco-Paulo A. (2007). Influence of mechanical agitation on cutinases and protease activity towards polyamide substrates. Enzyme Microbial Technol. 40, 1678–1685. 10.1016/j.enzmictec.2006.09.001

[B25] Socrates G. (2004). Infrared and Raman Characteristic Groups Frequencys – Table and Charts. John Wiley and Sons, Ltd.

[B26] Song J.E. Kim H.R. (2017). Improvement in nylon fabrics 'reactivity via enzymatic functionalization. J. Tex. Inst. 108, 155–164. 10.1080/00405000.2016.1160758

[B27] Vecchiato S. Ahrens J. Pellis A. Scaini D. Mueller B. Acero E. H. (2017a). Enzymatic functionalization of HMLS-polyethylene terephthalate fabrics improves the adhesion to rubber. ACS Sustain. Chem. Eng. 5, 6456–6465. 10.1021/acssuschemeng.7b00475

[B28] Vecchiato S. Skopek L. Jankova S. Pellis A. Ipsmiller W. Aldrain A. (2017b). Enzymatic recycling of high-value phosphor flame-retardant pigment and glucose from rayon fibers. ACS Sustain. Chem. Eng. 6, 2386–2394. 10.1021/acssuschemeng.7b03840

[B29] Wang C. Wang Z. Liu Z. (2018). Flame - retardant rigid polyurethane foam with a phosphorus - nitrogen single intumescent flame retardant. Polym. Adv. Technol. 29, 668–676. 10.1002/pat.4105

[B30] Wang Y. Chang Y. Yin L. Xue Y. Li Z. Xue C. (2016). A novel technological process of extracting l-tyrosine with low fluorine content from defatted antarctic krill (*Euphausia superba*) by-product by enzymatic hydrolysis. Food Bioproc. Technol. 9, 621–627. 10.1007/s11947-015-1658-x

[B31] Wang Y. Wang F. Dong Q. Xie M. Liu P. Ding Y. (2017). Core-shell expandable graphite @ aluminum hydroxide as a fl ame-retardant for rigid polyurethane foams. Polym. Degrad. Stabil. 146, 267–276. 10.1016/j.polymdegradstab.2017.10.017

[B32] Watanabe I. Sakai S. (2003). Environmental release and behavior of brominated flame retardants. Environ. Int. 29, 665–682. 10.1016/S0160-4120(03)00123-5 12850086

[B33] World Fire Statistics (2006). World Fire Statistics. International association of fire and rescue services (CTIF). Available online at: https://www.ctif.org/world-fire-statistics

[B34] Yang Z. H. H. Xiao Y. Zeng G. M. Xu Z. H. Y. Liu Y. S. H. (2007). Comparison of methods for total community DNA extraction and purification from compost. Appl. Microbiol. Biotechnol. 74, 918–925. 10.1007/s00253-006-0704-z 17115207

