# Supplementary material for: Increased Flame Retardancy of Enzymatic Functionalized PET and Nylon Fabrics via DNA Immobilization
Source: Front Chem. 2019 Oct 22;7:685. doi: 10.3389/fchem.2019.00685 (PMC6818624; doi:10.3389/fchem.2019.00685)
Supplement: Supplementary file 1 [file Data_Sheet_1.pdf]

Increased flame retardancy of enzymatic functionalized PET and nylon fabrics via DNA immobilization

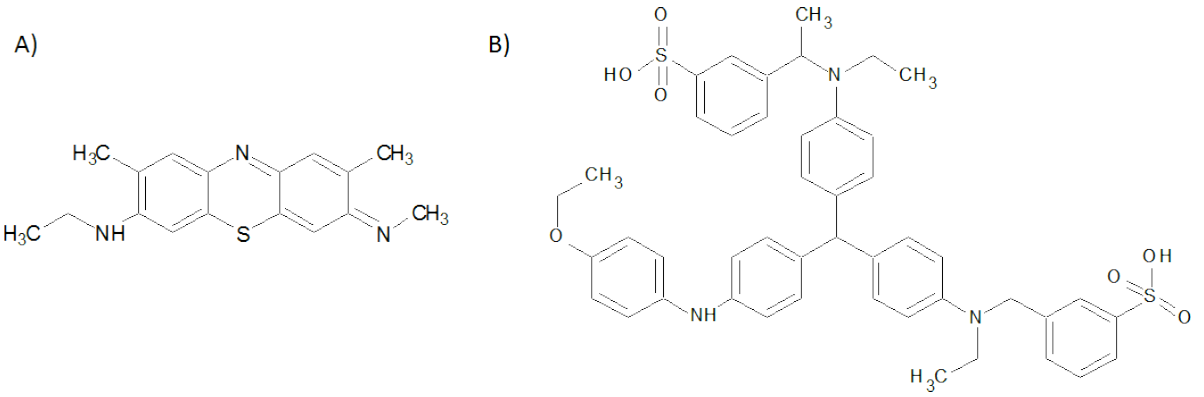

Figure 1: Chemical structure of Methylene Blue a) and Coomassie Brilliant Blue

Table S1: HPLC gradient for the measurement of PET hydrolysates

**Time [min]**	**A (H_2O + 0.1 % Formic Acid) [% v/v]**	**B (MeOH + 0.1 % Formic Acid) [% v/v]**
2	50	50
9	0	100
12	0	100
14	70	30

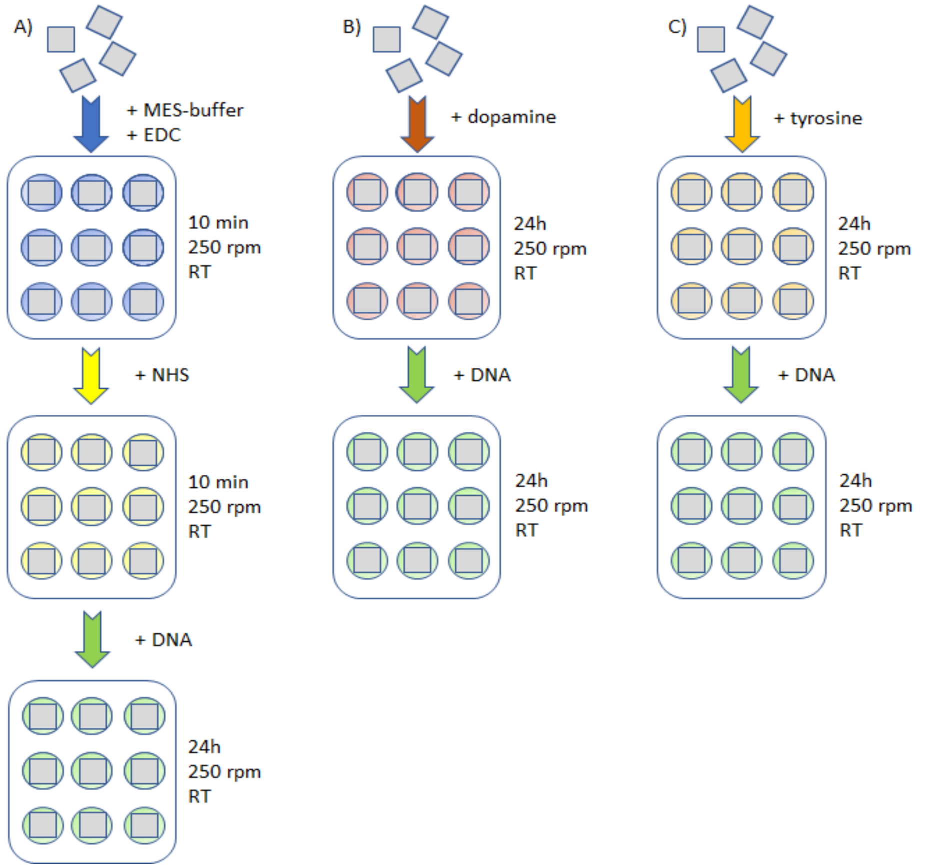

Figure 2 Schematization of DNA immobilization reactions

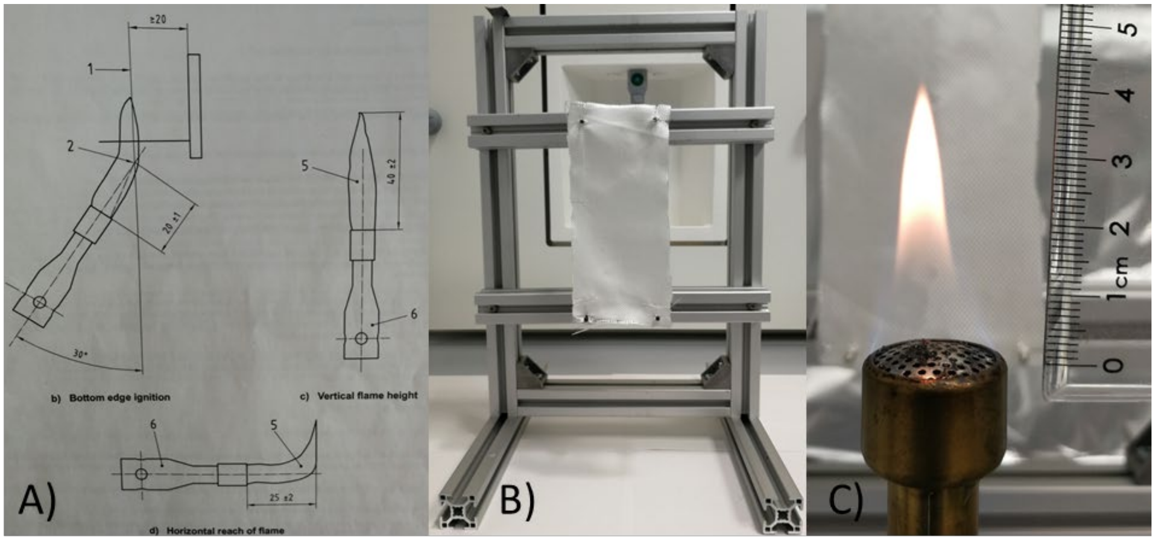

Figure 3 Flame position during bottom edge ignition, B) fixed fabric sample in the apparatus and C) flame of the burner set to a height of 4 cm

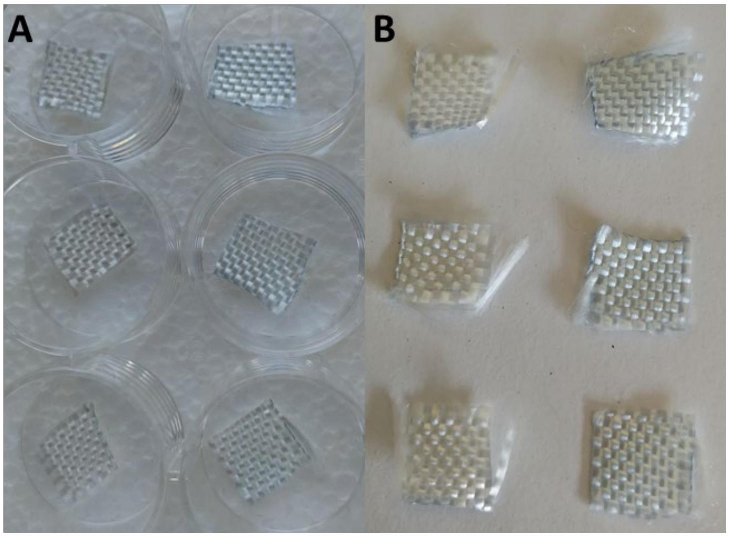

Figure 4: Samples after the treatment with acid and basic dye: A) PET blank (left) and enzymatic treated (right) after staining with Methylene blue; B) Nylon-6 blank (left) and enzymatic treated (right) after Comassie Brillant blue staining.

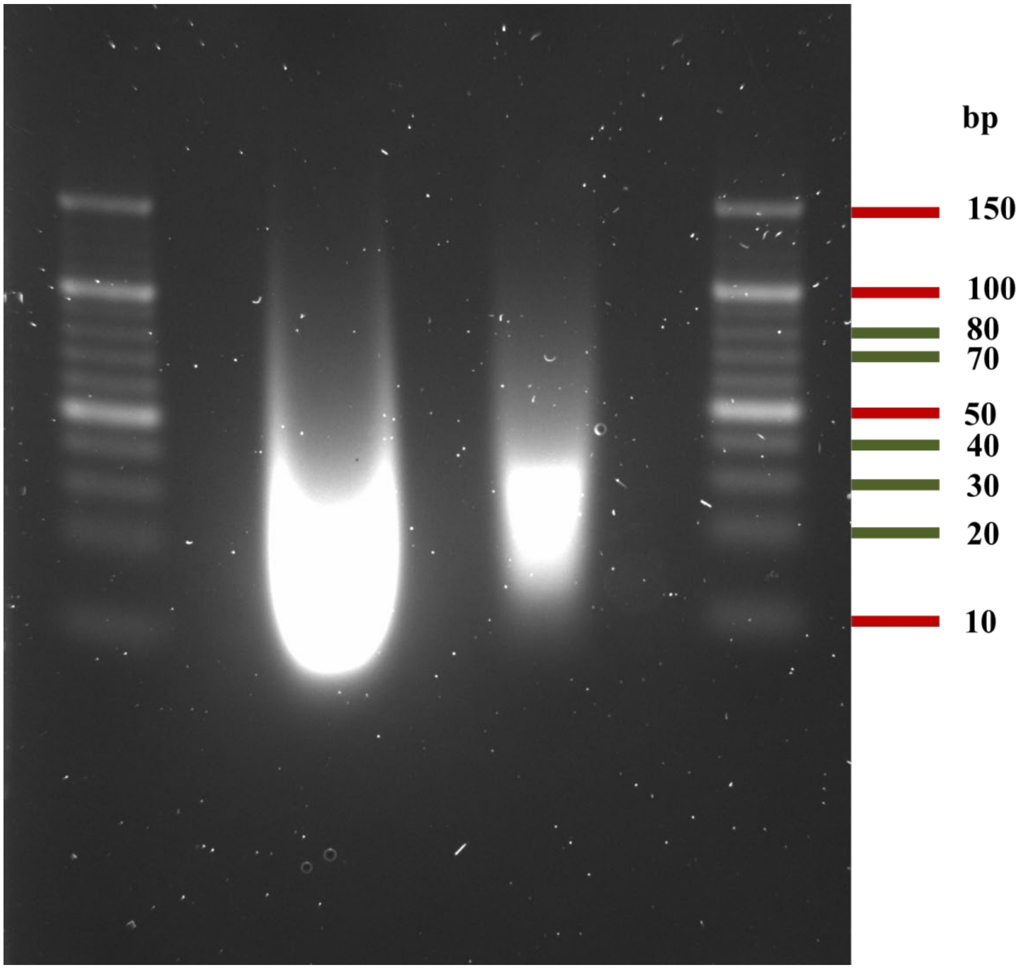

Figure 5 Agarose gel.

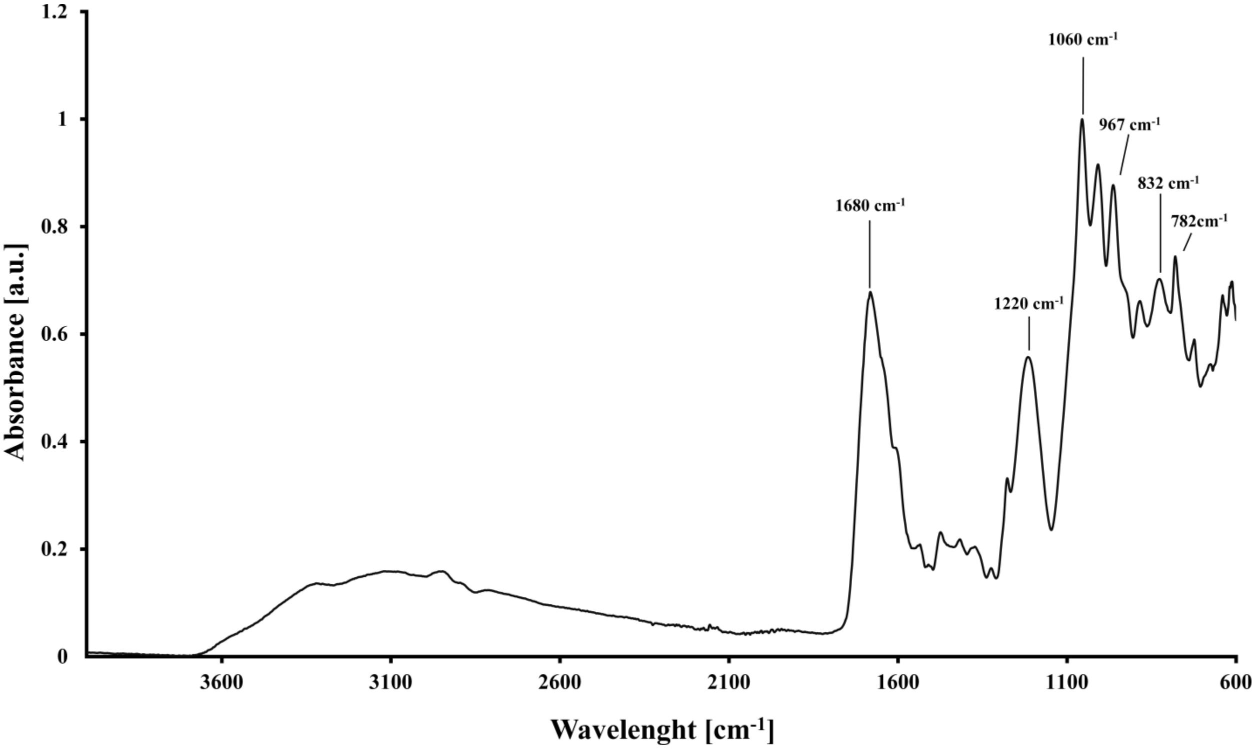

Figure 6: FT-IR of the DNA from salmon sperm.

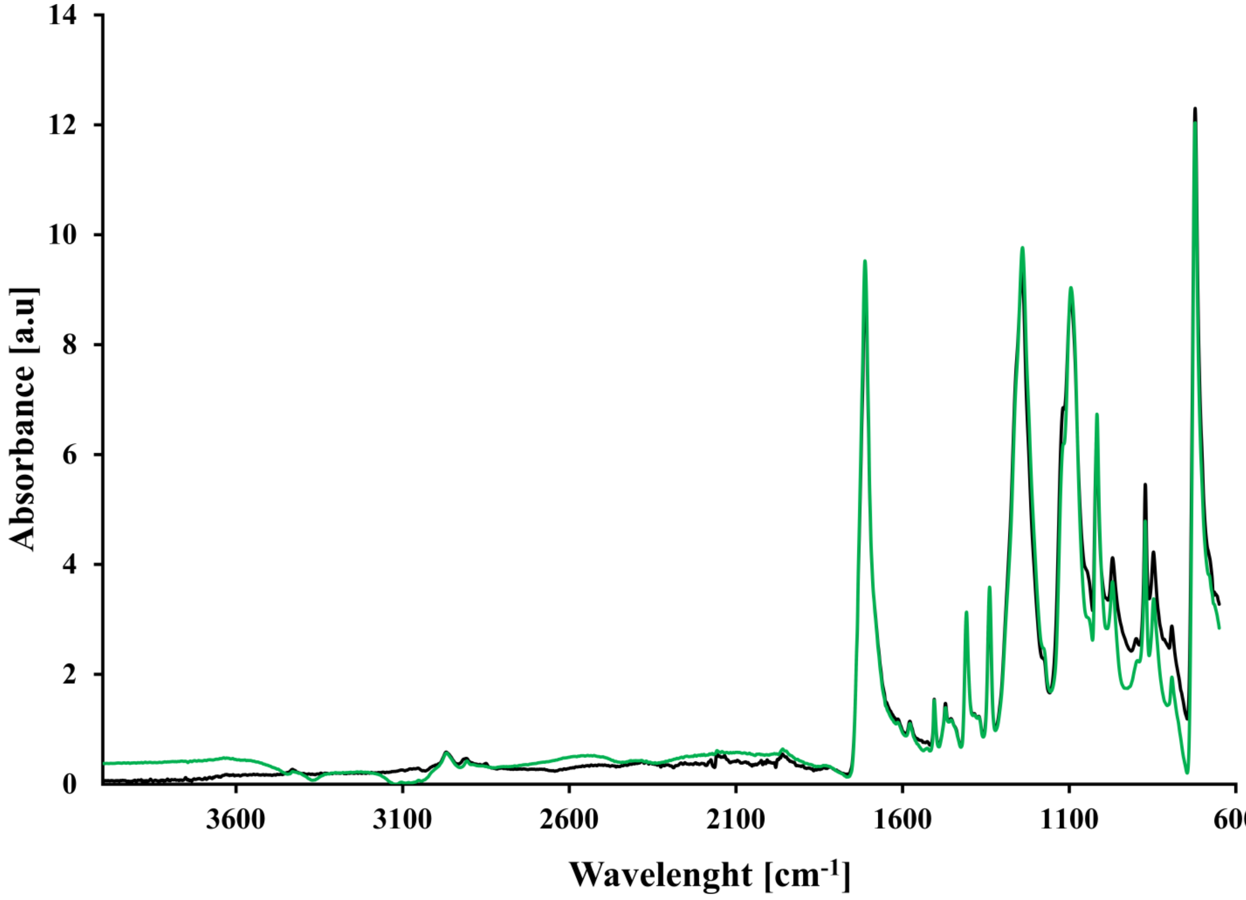

Figure 7: FT-IR of enzymatic treated PET (black line) and enzymatic treated PET coated with DNA via EDC/NHS (green line).

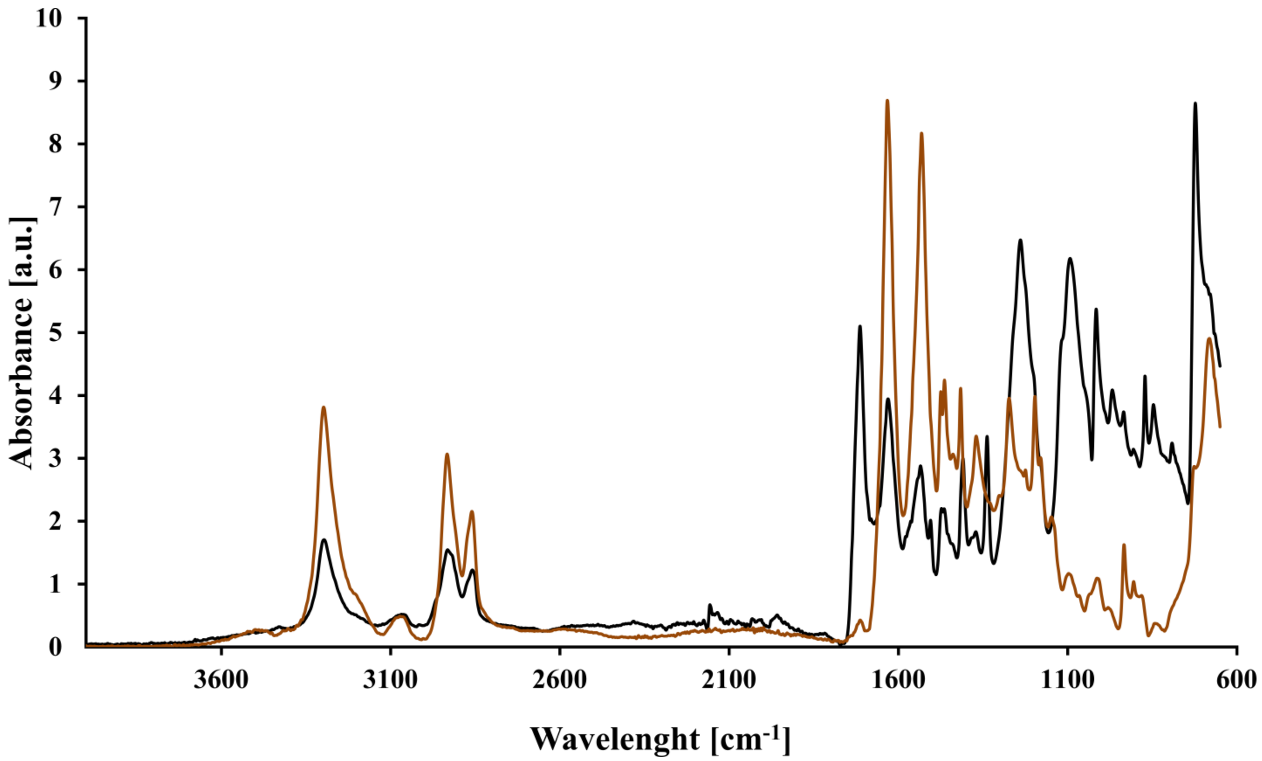

Figure 8: FT-IR of enzymatic treated Nylon (black line) and enzymatic treated Nylon coated with DNA via EDC/NHS (orange line).

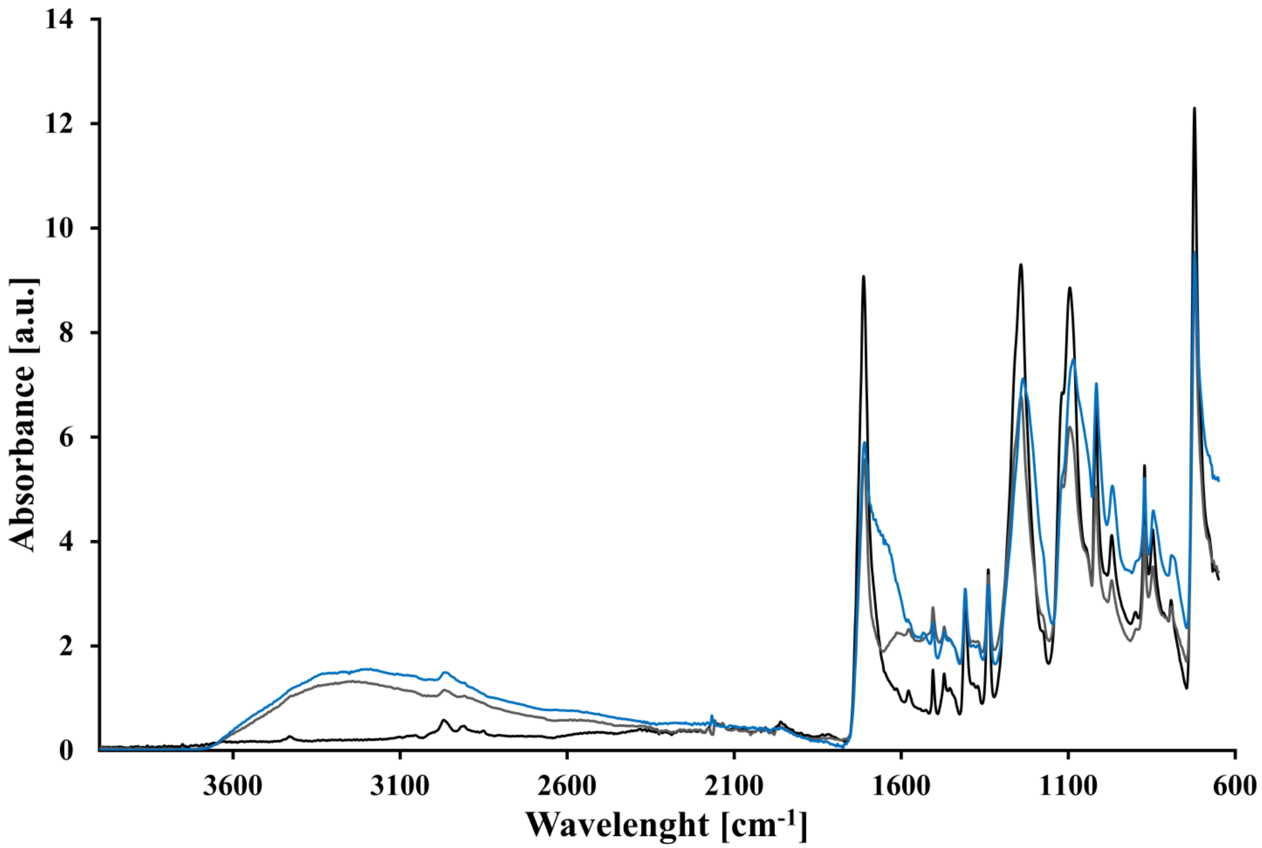

Figure 9: FT-IR of enzymatic treated PET (black line) and enzymatic treated PET coated with dopamine (grey line) and enzymatic treated PET coated with dopamine/DNA.

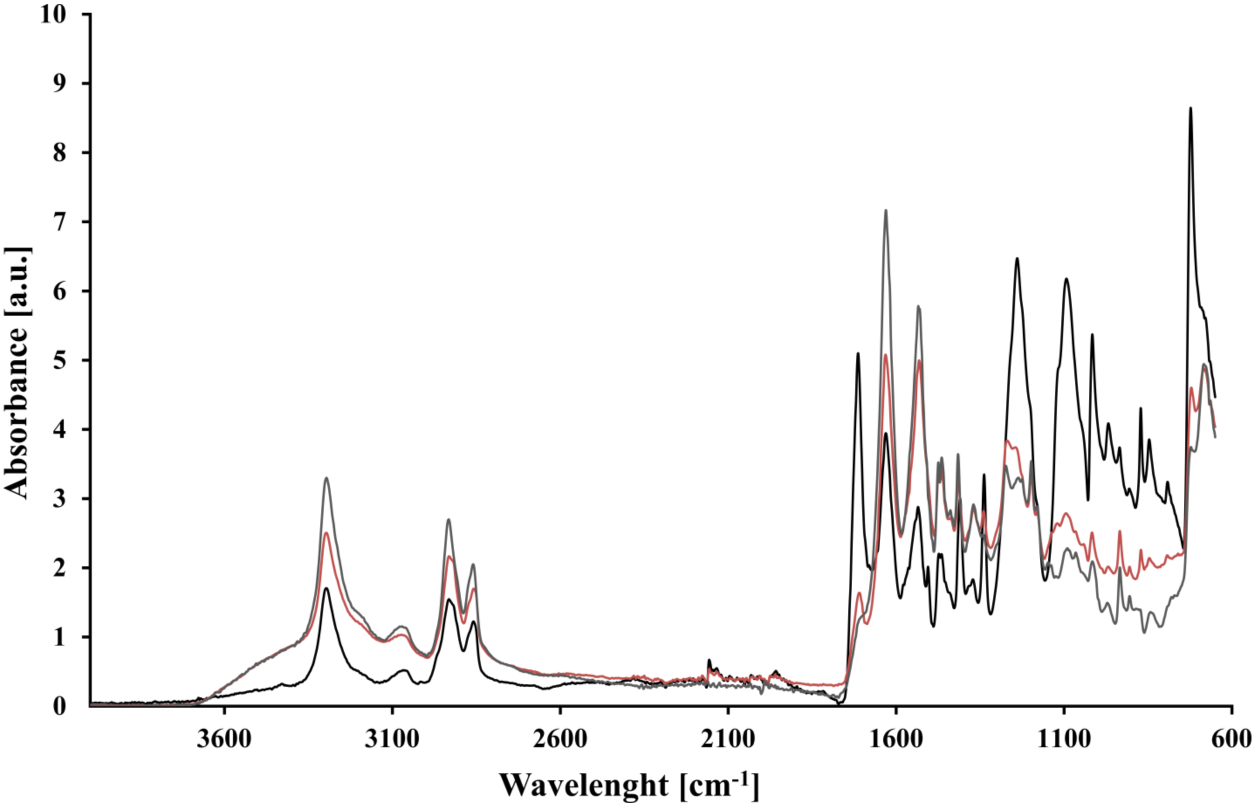

Figure 10: FT-IR of enzymatic treated Nylon (black line) and enzymatic treated Nylon coated with dopamine (grey line) and enzymatic treated nylon coated with dopamine/DNA.

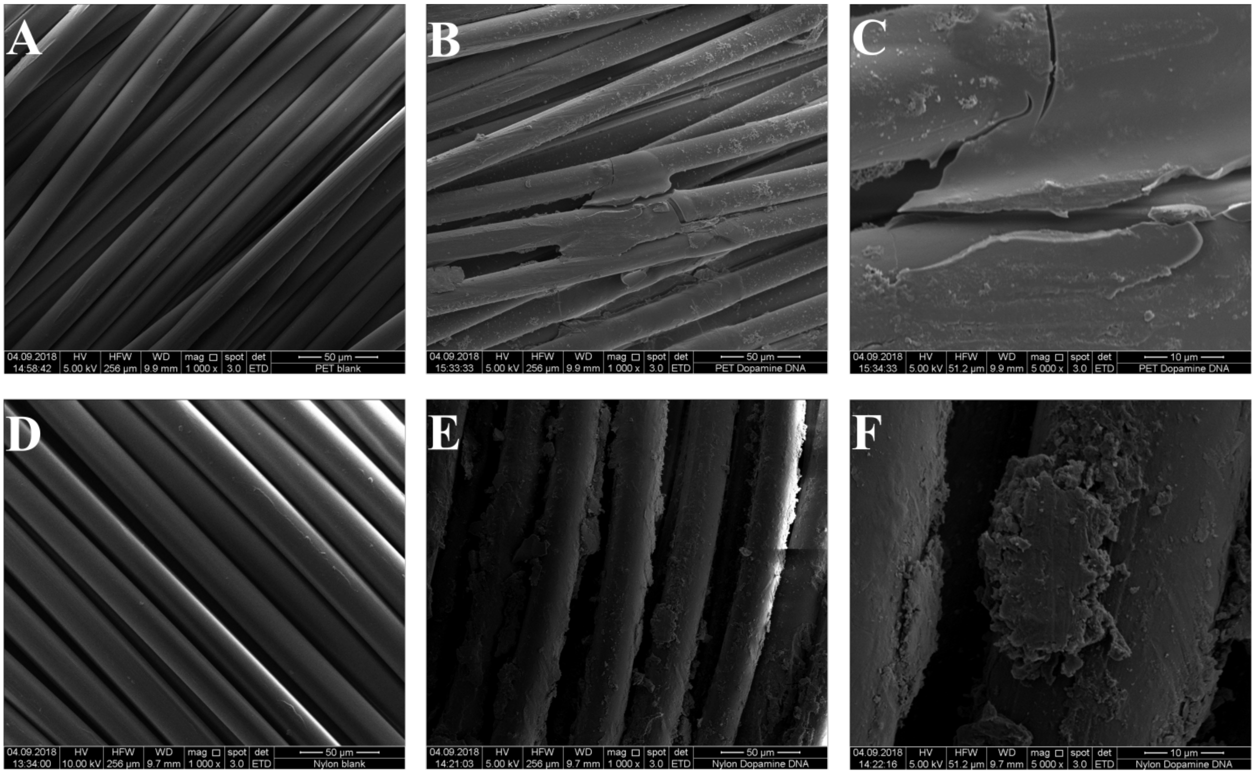

Figure 11: SEM pictures of **A)** untreated PET 1000x magnification **B)** PET_dopamine_DNA 1000x magnification **C)** PET_dopamine_DNA 5000x magnification **D)** untreated Nylon 1000x magnification **E)** Nylon_dopamine_DNA 1000x magnification **F)** Nylon_dopamine_DNA 5000x magnification.

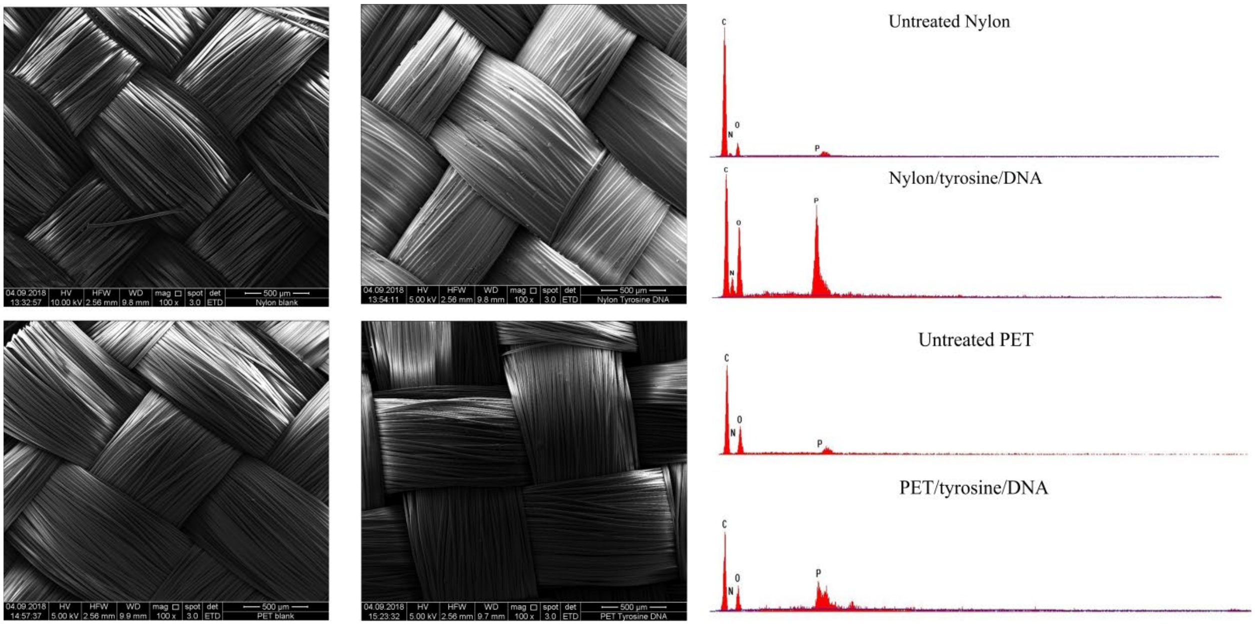

Figure 12: SEM pictures of **A)** untreated Nylon 100x magnification **B)** PET_tyrosine_DNA 100x magnification **C)** PET 100x magnification **D)** Nylon_tyrosine_DNA 100x magnification **E)** EDS results.

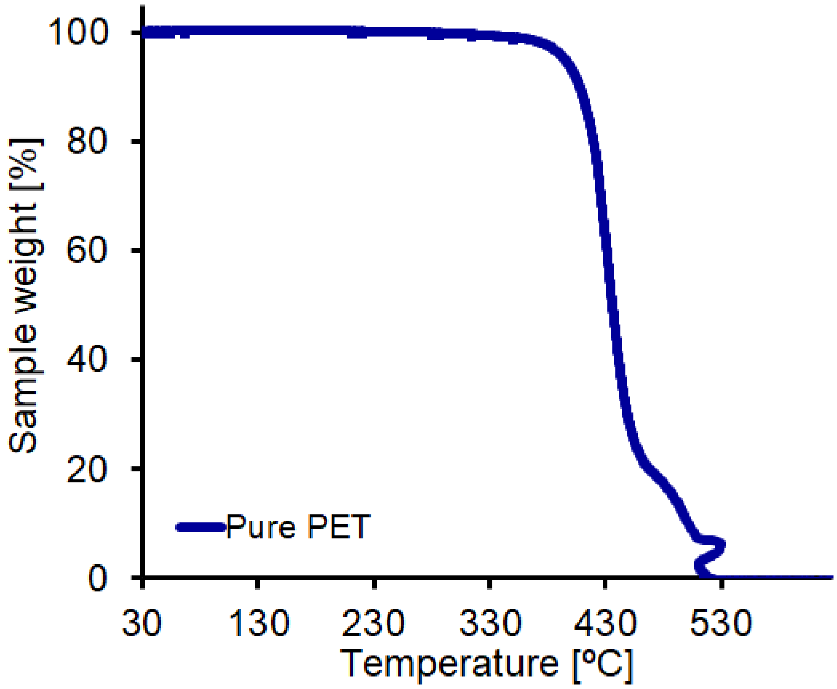

Figure 13. TGA analysis of pure PET using air as the gas.

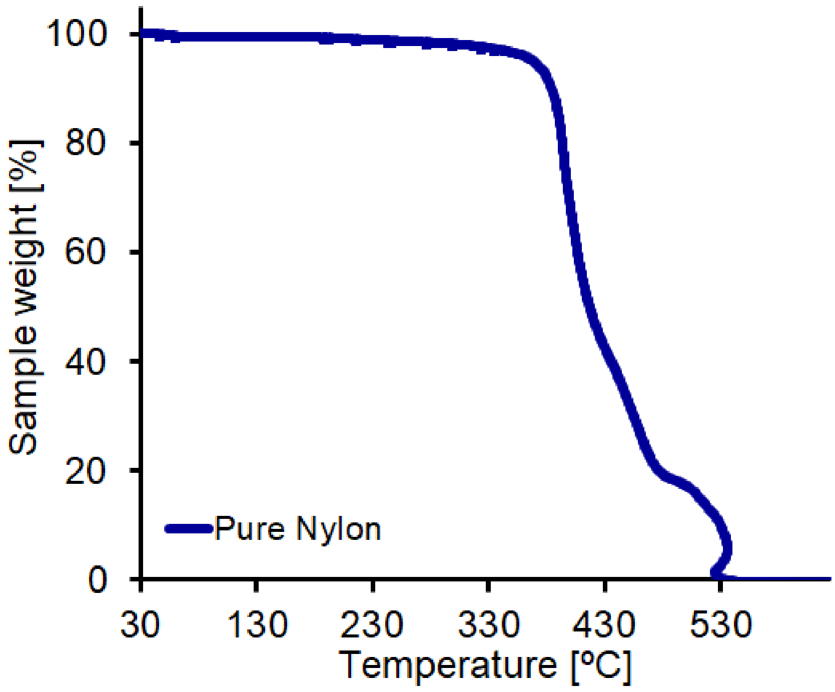

Figure 14. TGA analysis of pure Nylon using air as the gas.
